# The effect of tertiary treated wastewater on fish growth and health: Laboratory-scale experiment with *Poecilia reticulata* (guppy)

**DOI:** 10.1371/journal.pone.0217927

**Published:** 2019-06-11

**Authors:** Inbal Zaibel, Yuval Appelbaum, Shai Arnon, Malka Britzi, Frieda Schwartsburd, Shane Snyder, Dina Zilberg

**Affiliations:** 1 French Associates Institute for Agriculture and Biotechnology of Drylands, The Jacob Blaustein Institutes for Desert Research, Ben-Gurion University of the Negev, Midreshet Ben-Gurion, Israel; 2 Zuckerberg Institute for Water Research, The Jacob Blaustein Institutes for Desert Research, Ben-Gurion University of the Negev, Midreshet Ben-Gurion, Israel; 3 The National Residue Control Laboratory, The Kimron Veterinary Institute, Ministry of Agriculture, Beit Dagan, Israel; 4 Department of Chemical & Environmental Engineering, University of Arizona, Tucson, Arizona, United States of America; VIT University, INDIA

## Abstract

Treated wastewater (TWW) constitutes a sustainable water resource and has been used for fish culture in some countries around the world, although there are no comprehensive data on the effect of TWW on fish growth and health in the context of aquaculture production. Our objectives were to examine how fish culture in TWW affected fish growth and fitness, as well as compliance with the international standards for safe consumption. Guppy (*Poecilia reticulata*) fingerlings were reared in 0%, 50% and 100% tertiary TWW (TTWW), from the age of five days, for a period of four months. In water analyses, 33 out of 67 tested organic micropollutants (OMPs) were detected in the TTWW samples at least once, at concentrations that are typically reported in domestic TTWW. Fish survival ranged between 77–80% and did not differ between treatment groups. Fish growth and mortality following challenge infection with *Tetrahymena* sp. (which ranged between 64–68%), were similar among treatment groups. Of tested immunological parameters, lysozyme and anti-protease was similar among treatments while complement activity was highest in the 50% TTWW-reared fish. No abnormalities were observed in the histopathological analysis. Levels of heavy metals, polychlorinated-biphenyls (PCBs) and organochlorines (OCs) in fish were below the detection limit and below the Food and Agriculture Organization of the United Nations (FAO) and the European Union EU maximal permitted levels in food fish. Results suggest that the yield of fish grown in TTWW is potentially similar to that in freshwater, and the produced fish comply with the standards of consumer safety. The results are in line with previous studies that examined the feasibility of TWW-fed aquaculture.

## Introduction

Aquaculture is the fastest growing food production sector in recent years, largely due to the expanded consumption of fish as a preferred source of animal protein, alongside a relatively static level of capture fisheries [1] due to declining natural fish stocks [2]. Inland aquaculture could potentially increase the amount of fish production, further replacing natural fisheries. However, inland aquaculture has traditionally been water-source dependent, relying on nearby streams, lakes, springs, groundwater, etc. [3]. Therefore, water availability is already a major limiting factor in the expansion and growth of this fish production sector in arid regions. In addition, according to the food and agriculture organization (FAO, 2016, 2018) prediction for the early 2020s, is that aquaculture will cover only 40% of global fish demand. In order to fill this fish demand-supply gap aquaculture would need to globally grow in 9.9% every year. However, the annual growth rate of global aquaculture production is expected to decline (from 5.7% in 2003–2016 to 2.1% in 2017–2030). Availability and accessibility of good quality water are mentioned as main reasons for this slowdown [1,4].

One way to overcome water shortage is to seek alternative resources. Treated wastewater (TWW) constitutes a sustainable water resource that has been used for aquaculture throughout Asia and the Far East for centuries including China [5,6] and India [7,8]. This practice had also been applied in Germany in the past for over a century [9], and was examined experimentally in several countries, including USA [10,11], Hungary [12], Peru [13], Egypt [14], and Israel [15,16].

Reuse of TWW for aquacultural applications is not officially practiced or recommended in most countries in Western Europe and North America due to public health concerns. Yet, it offers a solution for many of the challenges that global freshwater aquaculture is facing. Indeed, TWW use for food production has been applied successfully and intensively in agriculture through crop irrigation [17]. TWW-fed aquaculture can be applied anywhere near wastewater treatment plants (WWTPs), including in arid and semi-arid climates, independent from natural water resources. In nearly all cases, WWTPs are located near urban density centers, thus offering the advantage of an important food commodity’s production close to the demand centers. In addition, TWW use can reduce both the demand for freshwater and water costs in existing aquacultural operations, thus rendering the fish production more profitable.

Recycling TWW for aquaculture does not come without concerns. The potential of microbial contamination in TWW-fed aquaculture was widely investigated [5,15,18]. Almost 20 years ago, Mara and Cairncross suggested in 1989 a tentative guideline value of <10^3^ fecal coliforms/100 mL of fish pond water [19], a value that was accepted by the World Health Organization (WHO) soon after [20]. Fewer studies exist on heavy metals bioaccumulation in fish reared in TWW [16,21,22]. According to these studies, fish culture in TWW was found to be safe, referring to the maximal permitted levels of heavy metals in food fish [23,24]. Another major concern that has gained a great deal of attention in recent years is the occurrence of organic micropollutants (OMPs) in TWW, and their potential effect on aquaculture has not been widely investigated. In nature, fish are exposed to TWW emitted from wastewater treatment plants and sewage treated to various levels, when the latter flows into natural rivers and streams. For example, polychlorinated-biphenyls (PCBs) have been shown to affect fish thyroid and steroid hormone systems, and elevated body concentrations of PCBs were correlated with a reduction of oocyte size in females and less developed testes in males [25]. Exposure to pharmaceutical residues led to a significant decrease in embryo production [26], and is also associated with the induction of stress behavior [27,28]. Endocrine-disrupting chemicals (EDCs) in TWW, such as hormones, are also a major concern because of their negative effect on natural fish populations [29,30]. Some of the negative effects that were attributed to EDCs include the occurrence of intersex conditions in male fish, altered levels of sex steroid hormones, and the induction of vitellogenin, the precursor of egg yolk, in male fish [31,32]. While fish exposure to OMPs has led to adverse effects from an ecological point of view, the impacts of this exposure to TWW and OMPs on fish growth, health, immune function and bioaccumulation have rarely been studied in terms of TWW reuse for aquaculture and are still largely unknown.

The question of whether TWW can be used for aquaculture comprises technological, scientific, social, and human health aspects. Here we addressed the basic questions of whether fish can grow well in tertiary TWW (TTWW) and whether the fish cultured in TTWW meet the international standards for edible fish [23,24]. The specific objectives of this study were to evaluate the effect of TTWW on fish growth and health status and to measure the bioaccumulation of heavy metals and PCBs in fish tissues. Guppies (*Poecilia reticulata*) were chosen as a model fish due to rapid development and short life cycle. These features enable the evaluation of TWW’s effects during the entire phase of the fish’s growth and development, from birth to maturity [33–36]. Therefore, using guppies as a model fish enabled to provide a proof of concept over a relatively short time period and modest budget. Although food fish are larger and their growth may take from 6 months to more than a year, accumulation of contaminants in fish muscle and other tissue can be detected after a very short exposure such as few weeks [27,37,38].

## Material and methods

### Experimental setup

Guppy (*Poecilia reticulata*) fingerlings at the age of one day were obtained from a commercial ornamental fish farm in southern Israel and maintained in a 100-L acclimation tank for five days. The acclimation tank and experimental aquaria were aerated, maintained in a temperature-controlled room at 24 ± 1.5°C and a photoperiod of 12:12 h, and supplied with submerged bio-filters (one 4-L bio-filter in each 30-L aquarium, and three such filters in the 100-L tank) to convert the ammonia excreted by the fish to the much less toxic nitrate, by nitrifying bacteria.

On day 6, fish (weight of 40 ± 19 mg) were stocked into the experimental 30-L aquaria at a density of 100 fish per aquarium, for a period of 123 days. The experimental aquaria were filled with three different types of water: 100% tap water (control, after adding 2 mg sodium thiosulphate per 1 L of water in order to neutralize chlorine residues from the routine chlorination that is applied to drinking water), 100% TTWW, and a mixture containing 50% TTWW-50% tap water, in four replicates (total of 12 experimental aquaria). The TTWW was taken from the Yeruham wastewater treatment plant (WWTP), Israel. This WWTP serves a population of 9,700 people and receives approximately 2000 m^3^/day of domestic and industrial wastewater. Wastewater in the Yeruham WWTP is treated to a tertiary level, including conventional extended activated sludge as a secondary treatment followed by sand filtration and chlorination as a tertiary treatment and disinfection. TTWW was collected weekly from the storage reservoir and transferred to the laboratory, where it was stocked in 100-L tanks and treated with a submerged aerated biological filter for at least one day before being added to the experimental aquaria in order to eliminate any remaining ammonia and nitrite. Aquaria were siphoned, and water exchange of approximately 17% each time was performed three times a week.

Guppies are live bearers, reaching sexual maturity within 2–3 months of age and the typical gestation period is 21–30 days. Fry are relatively large and have a wide enough mouth opening which allows immediate feeding with Artemia. The experimental feeding regime was based on a standard protocol used by the Aquaculture Department of Central and Northern Arava R&D (Yair Kohn, Nitzan Reiss-Hevlin, Central and Northern Arava R&D, Personal communications) and Sharon et al., 2014 [39], with modifications. Feed was applied three times a day throughout the study. Artemia (Great Salt Lake, Utah, USA) was used to feed the fish until day 9 at a rate of approximately 500 nauplii/fish/day. The Artemia amount was then reduced to 170 nauplii/fish/day that were given once a day until day 26 and dry commercial food (Ocean Nutrition, Belgium; size 0) was added at 10% of body weight (BW) per day (applied in three feedings). To reduce dispersion of the food in the aquarium and its contamination, the food was reshaped into small balls by mixing it with a small quantity of egg white. The food size was increased to 0.3–0.5 pellets on day 27 and applied at 8% BW/day. Feeding was gradually reduced from week 7 and onwards by 1% per week, until reaching 2% BW/day by week 12.

Elevated mortalities were observed in the aquaria on day 8, and the monogenean ectoparasite *Gyrodactylus turnbulii* (commonly found on gills, skin and fins) was diagnosed as the cause. During the outbreak, humane endpoint euthanasia was applied by immersion in 250 μL/L clove oil. The fish were observed three times a day and any moribund fish (characterized by lethargic and/or uncoordinated swimming) were immediately removed and euthanized, but there were some mortalities that occurred over night with young fingerlings. The infection was treated in all aquaria with Praziquantel (Vetmarket, Israel) at a concentration of 3 mg/L for 4 h, followed by 50% water exchange. Treatment was repeated seven days later.

Fish were treated in compliance with the principles for biomedical research involving animals, and the experimental protocol was approved by the Ben-Gurion University of the Negev Committee for the Ethical Care and Use of Animals in Experiments (ethic authorization no. IL-34-05-2015). Humane endpoint euthanasia was applied to sick and lethargic fish following controlled infection (*Tetrahymena*, as described below), or un-intended diseases outbreak (*G*. *turnbulii*, as described above). Fish were anesthetized with 250 μL/L of clove aromatic oil (Frutarom, Israel) prior to sampling.

### Fish sampling, growth measurements, and determination of the hepato-somatic index

Fish were weighed on days 0, 23, 30, 37 and 51 to evaluate growth rates. Due to the small fish size, measuring individual weight was not feasible, and therefore, pools of fish were weighed together. Weight was determined at stocking and during the trial by randomly catching 40 fish from each aquarium and weighing them in two batches of 20 fish each, a subset of the original 100 fish in the aquarium. Weighing was carried out using precision balances (BJ 2200C, Precisa Gravimetrics, Switzerland). Weighing was ceased after day 51 when signs of sexual maturity began to appear [40], including increase in anal fin and coloration in males; and in females changes in body shape, *i*.*e*. distention and rounding of the abdomen and the occurrence of dark gravid spot near the anal vent. As guppies sexually mature at 2–3 months, it is logical that sexual differentiation will start to occur at about 51 days. Since of this point, the fish invest in developing their sex organs; thus somatic growth is interrupted, and large differences between genders begin to emerge [40,41]. The next weighing took place at experimental termination on day 123, when individual weight was determined, and males were separated from female fish.

Upon experimental termination (day 123), all the fish from the experimental aquaria were sampled, except for 20 fish, which were left in their original aquaria and used for a challenge infection with the protozoan parasite *Tetrahymena* sp. The sampled fish were individually weighed and collected for various analyses, including immunological assays—lysozyme, alternative complement pathway (ACH50) and antiprotease activity (8 fish per aquarium, 32 per treatment), and histopathology (10 fish per aquarium, 40 per treatment). All the aforementioned fish samples included 50% males and 50% females. The remaining fish were used to determine the hepato-somatic index (HSI) and to analyze bioaccumulation of heavy metals and PCBs. For HSI, livers were carefully separated from the rest of the body; the liver and body were then weighed in pre-weighted aluminum foils, immediately placed in liquid nitrogen, and then stored in -80°C until further analysis. HSI was calculated as the liver weight/whole body weight × 100.

### *Tetrahymena* challenge infection

Twenty fish (10 males and 10 females) from each aquarium were kept in their original aquarium for an additional 32 days under the same culture conditions and water treatments and then moved to 10-L infection aquaria (with similar conditions to the 30-L aquaria). Fish were anesthetized and infected with *Tetrahymena* by intraperitoneal (IP) injection of 1000 ciliates in 5 μL of an RM9 medium [42] using a 30-G needle fitted to a 100-μL syringe attached to an automated micro-injection chamber (model fusion 720, Chemyx Inc. Stafford, Texas, USA). This infection level was shown to be the LD50 infection dose [43]. Mortalities were recorded daily for 15 days after the injection. Wet mounts of skin and internal organs were collected from dead fish by scrapping the skin with a cover slip and collecting internal organs from dissected fish with scissors (including liver, spleen, kidney). These were placed on a microscope slide with a drop of water and immediately examined microscopically to confirm infection with *Tetrahymena* sp.

### Immunological analyses of whole-body homogenates

The immune parameters analyzed included lysozyme, alternative complement pathway (ACH50) and antiprotease activity. During sampling, the intestine, gall bladder and tail fin were removed, while the remaining body was weighed, snap-frozen in liquid nitrogen, and stored at -80°C. Whole body homogenates (WBH) were prepared by grinding frozen fish with a mortar and pestle, along with the constant addition of liquid nitrogen. Sodium phosphate buffer (0.05 M, pH 5.8) was added to the ground samples at a ratio of 2 mL of buffer per 1 g of sample and centrifuged for 30 min at 16,000 × *g* at 4°C. The supernatant was carefully removed with a glass Pasteur pipette, avoiding the overlying lipid layer. Samples were then aliquoted according to the volume needed for each analysis and frozen at -80°C until analysis.

Lysozyme: Lysis of the *Micrococcus lysodeikticus* bacterium by lysoplate assay was modified from Nayak et al. [44] and Lie et al. [45]. Briefly, a homogenized suspension of 1% agarose in phosphate citrate buffer (0.1 M pH 5.8) containing 150 μg/mL *M*. *lysodeikticus* was poured into petri dishes (diameter of 10 cm). After solidifying at room temperature, the plates were maintained at 4°C for up to five days before use. Wells were perforated in the agar plates by drawing the agar with a 3 mm-diameter plunger. Standards were prepared with chicken egg white lysozyme (Sigma, L-6876, EC 3.2.1.17) in PBS (0.05 M, pH 6.2) at 10 different concentrations, ranging from 3.9–200 μg/mL. Samples of WBH were concentrated by five, by complete freeze-drying and re-hydration with double-distilled water (DDW) at the required volume. Then, 8 μL of standards and concentrated samples were added to the wells in triplicate and incubated at 22°C for 24 h. After incubation, the diameter of the clear zone around the wells was measured, and concentrations were determined as the proportion of standards diameter to the log of their lysozyme concentration.

The alternative complement pathway (ACH50) assay was carried out conforming to Sunyer and Tort [46], with some modifications. The fish’s complement system was challenged by rabbit red blood cells (RRBCs) in the presence of gelatin veronal buffer (GVB) containing ethylene glycol tetraacetic acid (EGTA). Samples of WBH were tested at eight different concentrations ranging from 1:512 to 1:65,536 (double dilutions in EGTA-GVB). A total of 100 μL of the WBH sample and 30 μL of the RRBC suspension (4 × 10^8^ cells/mL) were added to each 1.5 mL Eppendorf tube. A blank was obtained by adding the RRBCs to EGTA-GVB (instead of the sample), and 100% lysis was obtained by mixing 30 μL of RRBCs with DDW, and the analysis was carried out in triplicate. All samples and controls were incubated at 23°C with gentle shaking for 2 h. The hemolytic reaction was terminated using 1000 μL of buffer containing 10 mM EDTA followed by centrifugation at 1600 × *g* for 10 min at 4°C. Finally, the reaction quantification was determined according to the optical density of the supernatants using a Tecan Sunrise microplate reader (Salzburg, Austria) at 414 nm. Results were presented as the number of ACH50 units/mL of WBH that provided 50% lysis, calculated according to Sunyer and Tort [46].

The measurement of antiprotease activity in WBH was performed according to Magnadottir [47]. First, 20 μL of each WBH sample was incubated with 20 μL of trypsin (5 mg/mL) in a 1.5-mL Eppendorf tube for 5 min. Then, 250 μL of azocasein (protease substrate; at 20 mg/mL) and 200 μL PBS were added and incubated at 24°C for 1 h. The reaction was terminated by adding 500 μL of 10% tricarboxylic acid solution followed by another incubation at the same temperature for 30 min. The samples were then centrifuged at 6000 *g* for 5 min, and 100 μL of the supernatants were added to a 96-well plate along with 100 μL of 1N NaOH in triplicates. Absorbance was measured at 405 nm with a Tecan Sunrise microplate reader (Salzburg, Austria). A negative control (blank) for the analysis was prepared by adding PBS instead of the WBH sample and trypsin (only PBS), and blanks for each sample were prepared by adding PBS instead of trypsin (sample + PBS). Maximal proteolytic activity (100% lysis) as a positive control was obtained by mixing PBS (instead of the sample) with trypsin. The percentage inhibition of trypsin activity for each WBH was calculated using the following equation:

$$\%\;\text{inhibition}=\left\{\frac{\text{OD}\;100\;\%\;\text{lysis}\;–\;\left(\text{OD of sample-OD of sample blank}\right)}{\text{OD of 100}\;\%\;\text{lysis}}\right\}\times 100$$

OD = optical density

### Histopathology

Histopathology was carried out on the fish sampled following the 123 days exposure to the different water treatments and prior to infection with *Tetrahymena*, as described above. A cut in the abdomen was performed in whole anaesthetized fish, the tail fin was cut off, and the body was immediately fixed in neutral buffered formalin. After 48 h, samples were transferred to 70% ethanol, where they were kept until analysis. Prior to processing the samples, they were placed in a decalcification solution (44% formic acid and 12.5% sodium citrate) for 12 h, rinsed in flowing tap water for 2 h, and returned to 70% ethanol. Processing was operated in a microwave histo-processor (RHS-1, Milstone, Italy), after which the samples were embedded in paraffin blocks and sectioned at 4 μm. Finally, the sections were stained with hematoxylin and eosin (H&E). Samples were analyzed under a light microscope, with special attention given to liver pathology and gonad development, as these were reported to be altered in fish following exposure to wastewater and associated contaminants in previous publications [48–51]. Liver pathology analysis included quantification of the occurrence of melanomacrophage centers (MMCs). Direct microscopic observation was used to calculate the number of MMCs per liver area. For this, the number of MMCs per liver section was counted, a snapshot of the specific liver section was taken, and the liver area was calculated using ImageJ 1.5n software. Gonads pathology analysis included microscopic observations to identify sperm cells or eggs at all developmental stages, and to search any evidence of intersex phenomena in order to examine any possible effects of OMPs and other compounds originated from the TTWW on sexual development.

### Chemical analyses in fish and water samples

Fish from the same aquarium were pooled together (whole bodies excluding the livers), ground using a mortar and pestle with constant addition of liquid nitrogen and kept at -80°C in amber glass jars. All tools and jars were pre-washed with nitric acid (10%) and acetone. Analyses of the four regulated heavy metals (Cd, Pb, As, and Hg), according to the Israeli Ministry of Health, were performed as follows: 0.5 gr of a tissue sample was digested with 4 mL of nitric acid (65%) in a special Teflon vial for 14 h at 80°C in an oven (Heratherm OMS60, Thermo Fisher Scientific Inc., UK). After digestion, the samples were brought to room temperature and then transferred into polypropylene 15 mL test tubes and diluted to 5 mL with bi-distilled water (Micropure, Thermo). Analysis of As and Pb was performed with no further treatment, while for Cd analysis, the samples were diluted by twofold. For Hg measurements, 0.2 mL of the digested sample was mixed with 9.8 mL of bi-distilled water and 20 μl of a KnMO4 5% solution.

The concentrations of Cd, Pb, and As in the fish samples were measured with an atomic absorption graphite furnace system AA 600 (Perkin-Elmer, USA) utilizing a palladium matrix modifier. A mercury analyzer, FIMS 100 (Perkin-Elmer), was used to determine the Hg concentration. The concentrations in the samples were calculated by utilizing a matrix-matched calibration curve. Blank fish tissue was fortified with the four elements prior to the digestion step and then treated as described earlier. The fortification concentrations were 500, 1000, 2000 μg/gr for As; 25, 50, 100 μg/gr for Cd; 150, 300, 600 μg/gr for Pb; and 250, 500, 1000 μg/gr for Hg. The limits of quantification (LOQ) were 200 μg/Kg, 25 μg/Kg, 100 μg/Kg, and 100 μg/Kg for As, Cd, Pb and Hg, respectively.

Pooled fish samples were also analyzed for PCBs and OC pesticides. PCB analytes included PCB 28, PCB 52, PCB 101, PCB 118, PCB 153, PCB 138, and PCB 180. The OC pesticides included hexachlorobenzene, hexachlorocyclohexanes (β-HCH, γ-HCH), DDTs (p,p′-DDT, p,p′-DDE, p,p′-DDD and o,p′-DDE), heptachlor, aldrin, endosulfane (I and II), endosulfan sulfate, endrin, nitrofen, dieldrin, and chlordanes (α and γ).

A modified QuEChERS (quick, easy, cheap, effective, rugged and safe) AOAC (Association of Analytical Communities) International sample preparation approach was used for extraction and clean-up of PCB congeners and OC pesticides in fish tissue. First, 5 gr of fish tissue was weighed in a clean polypropylene 50-mL centrifuge tube, followed by the addition of 10 mL of bi-distilled water and homogenization by a benchtop generator (Polytron PT 10–35 GT, Kinematica, USA). Afterwards, tetrachloro-m-xylene and decachlorobiphenyl were added to each sample as surrogates. Then, 12 mL of an acetonitrile/ethyl acetate mixture (3:1 v/v) and 0.1 mL of acetic acid were added, and the sample was vortexed. Extraction salts (4 gr MgSO4, 1 gr sodium acetate; United Chemical Technologies, PA, USA) were added to each tube. The samples were then vortexed and centrifuged; next a 10-mL aliquot of the upper layer was transferred to a polypropylene dispersive SPE 15 mL tube containing 900 mg MgSO_4_, 150 mg C18EC, and 150 mg PSA (Agilent, USA). The dSPE tubes were then vortexed and centrifuged. The extracts were concentrated to 1 mL under a stream of nitrogen at 60°C, transferred to GC vials, and subsequently analyzed by gas chromatography with a micro-electron capture detector (GC- μECD, 7890 A, Agilent, USA).

Matrix-matched calibration curves were prepared by spiking blank fish tissues with standard mixtures of PCBs and OC pesticides after the homogenization step, which were then extracted and cleaned-up as described previously. The fortification levels were 50, 100 and 200 μg/Kg for PCBs, and 15, 30, and 60 μg/Kg for OC pesticides. The LOQ for PCBs was 5 μg/Kg, whereas the LOQ of OC pesticides was 1.5 μg/Kg.

A general water quality analysis was done once a week, including total ammonia nitrogen (TAN), nitrite and nitrate measurements using commercial kits (Merck, Darmstadt, Germany). In addition, electrical conductivity (EC), pH, dissolved oxygen (DO) and water temperature were measured with specific sensors connected to a handheld meter (WTW Multi 3400i, Weilheim, Germany). In addition, water samples were collected after 9, 39, 53 and 66 days during the experiment for analysis of OMPs. Freshly collected TTWW and tap water were placed in pre-washed (10% nitric acid and acetone) amber glass bottles. Sodium azide (1 gr/L) and ascorbic acid (100 mg/L) were immediately added to all samples to inhibit microbial activity and as a quenching agent, respectively. Samples were then frozen and kept at -20°C until shipment in frozen conditions to the University of Arizona (USA) for analysis. Water samples were analyzed for 67 OMP compounds, including steroid hormones, pharmaceuticals and personal care products (S1 Table). Samples were processed by on-line solid phase extraction (OSPE) and by direct injection mass spectrometry [52,53]. Briefly, each sample was spiked with an isotopically labeled surrogate standard mixture, followed by filtration with 0.2 mm syringe filter (Captiva polyethylene styrene (PES), Agilent Technologies, Santa Clara, CA, USA) for OSPE, and for direct injection. Both methods were coupled with liquid chromatography–tandem mass spectrometry (LC–MS/MS).

### Statistical analysis

Statistical analyses were performed at a probability level of 0.05. Survival rates and mortality following infection with *Tetrahymena* were calculated and compared using the Kaplan-Meier (Gehan-Breslow) survival analysis. Growth rates were compared using the MANOVA test. Final BW, HSI, and immunological assays, and MMCs/liver area were compared between the different treatment groups using ANOVA. A nested ANOVA (each individual aquarium was considered as a block, which was nested in the water treatment) was performed using values of each individual fish after omitting outliers [based on the acceptable methods of Z-score ($Z_{i}=\frac{Xi-\mu }{\sigma }$; range of 3 standard deviations) and numeric outlier calculation (X_i_ < Q1-1.5IQR, X_i_ > Q3+1.5IQR; where X is the actual value, Q is the quartile and IQR = Q3-Q1,)], and a data transformation was applied when needed (in cases in which the normality test failed). When the assumptions of the ANOVA were violated for an individual’s data, an ANOVA was performed for averages of each aquarium. Post hoc tests were performed with the Tukey HSD (for unequal n) or the Bonferroni test. All computations were performed with SigmaPlot 13.0 software and Dell Statistica 12. Full details and findings regarding the statistical analyses are available in supporting information S1 File.

## Results and discussion

### Water chemistry

The levels of TAN, nitrite, nitrate, pH, EC, and oxygen did not vary significantly over time within each water treatment group. The EC levels were, on average, 466 ± 32, 832 ± 61, and 1199 ± 76 μS/cm in tap water, 50% TTWW and 100% TTWW, respectively; and the pH was, on average, 6.95 ± 0.41, 7.22 ± 0.37, and 7.51 ± 0.41 in tap water, 50% TTWW and 100% TTWW, respectively. The differences between the treatment groups were not expected to affect the growth or the health status of guppies, being a euryhaline species with an ability to adjust to changing environments [54,55]. It is important to consider the fact that TWW may contain ammonia and nitrite in levels that can be stressful for fish, and therefore pre-treatment to eliminate these by bio-filtration may be required, as performed in the current study. A total of 33 of the 67 OMP compounds screened were detected at least once in the TTWW (Table 1). The detected compounds and concentrations found in this study are typical of those reported in municipal TTWWs, as found in several studies [52,53],[56–59].

**Table 1:** Categories, use, detection frequency, range and median (ng/L) of OMPs in four TTWW samples.

Category	Compound	Use	Detection frequency (%)	Range (ng/L)	Median (ng/L)
Pharmaceuticals	Atenolol	Beta-blocker	100	146–289	193
	Propranolol	Beta-blocker	100	15–37	22
	Sulfamethoxazole	Antibiotic	100	228–264	240
	Trimethoprim	Antibiotic	100	49–105	64
	Iohexol	Contrast agent	100	450–1300	790
	Iopromide	Contrast agent	100	175–3750	634
	Carbamezapine	Anti-seizure	100	1050–1810	1455
	Diclofenac	Anti-arthritic	100	82–729	641
	Diphenhydramine	Antiarrhythmic	100	116–658	603
	Diltiazem	Anti-histamine	50	19–43	31
	Hydrochlorothiazide	Antihypertensive	100	219–788	763
	Meprobamate	Anti-anxiety	100	24–76	65
Personal care products	Acesulfame	Artificial sweetener	100	261–8860	5070
	Sucralose	Artificial sweetener	100	9030–16800	15000
	Benzophenone	Sunscreen agent (UV blocker)	75	93–196	185
	Caffeine	Stimulant	100	104–730	420
Natural steroid hormones	Estrone	Estrogen	100	0.6–2.6	2
	17β-estradiol	Estrogen	100	0.9–1.6	1.05
	Progesterone	Progesterone	100	1.2–2.2	1.95
	17-Hydroxyprogesterone	Progesterone	25	3	3
	Testosterone	Androgen	100	2.2–3.8	3.05
Synthetic steroids	Betamethasone	Corticosteroid	100	1.4–2.3	1.55
	Deoxycorticosterone acetate	Corticosteroid	75	2.2–12.4	4.9
	Fluocinolone acetonide	Corticosteroid	100	1.5–64.4	19
	Fluticasone propionate	Corticosteroid	75	1.1–2.9	2.7
	Hydrocortisone	Corticosteroid	100	0.3–0.5	0.45
	Prednisone	Corticosteroid	100	2.9–5	3.85
	Triamcinolone acetonide	Corticosteroid	100	1.8–87.5	13.5
Industrial chemicals	TCEP	Flame retardant	100	153–189	176
	TCPP	Flame retardant	100	1750–4310	2440
	Bisphenol A	Plasticizer	100	8.5–740	28
	Benzotriazole	Corrosion inhibitor	100	238–448	397

### Survival and growth

**Fig 1:**
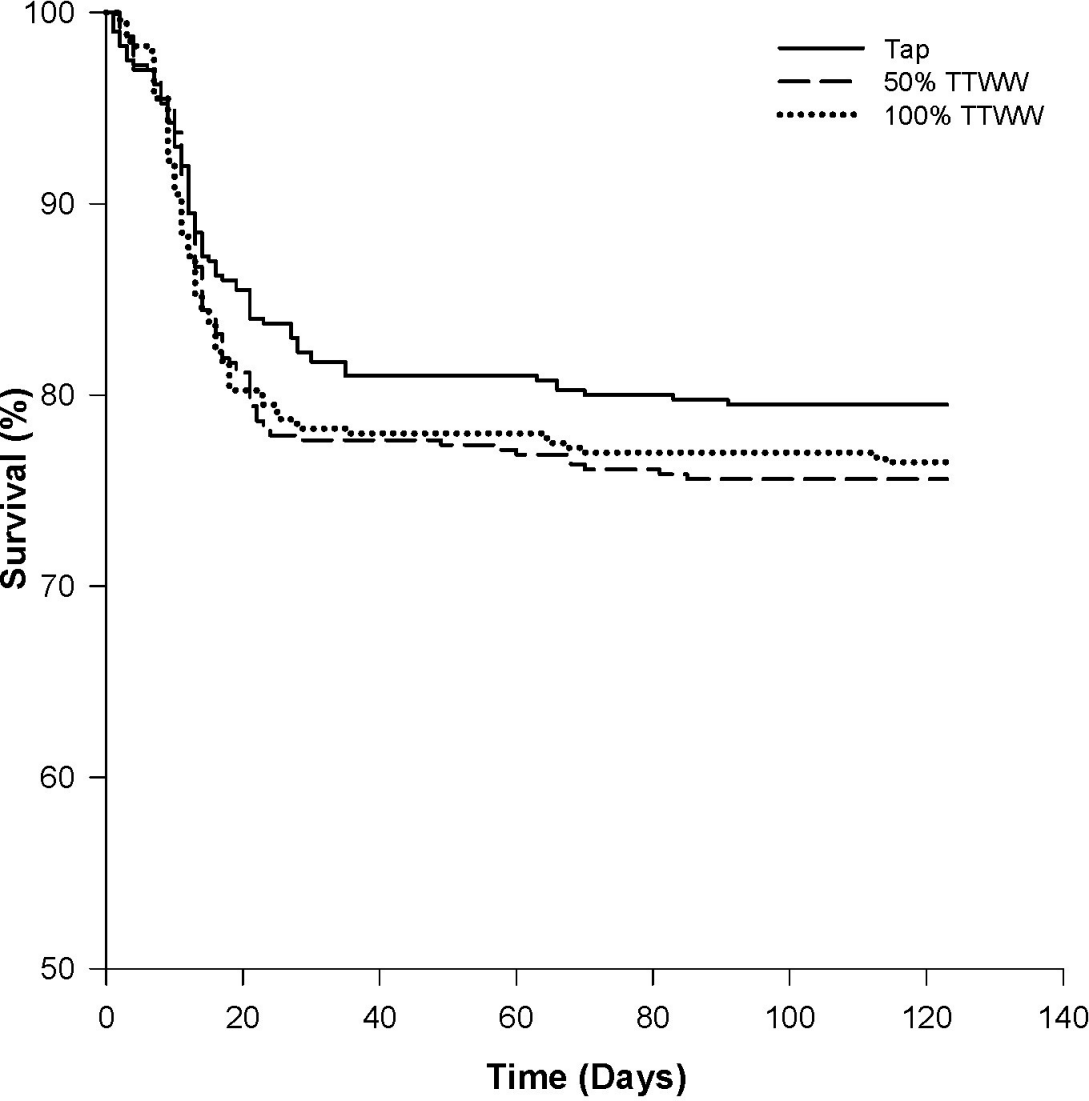
Mean survival rates of guppies exposed to different types of water during four months of growth.

Average survival rates in tap water, 50% TTWW and TTWW were 80 ± 2%, 76 ± 3% and 77 ± 4%, respectively, and no statistical differences were found between the different treatment groups (Fig 1). High mortalities were recorded starting from around day 8 in all treatment groups, and the monogenean parasite *Gyroductylus turnbulii* was identified as the causative agent. Treatment was applied, and the mortalities subsided (on day 21).

**Fig 2:**
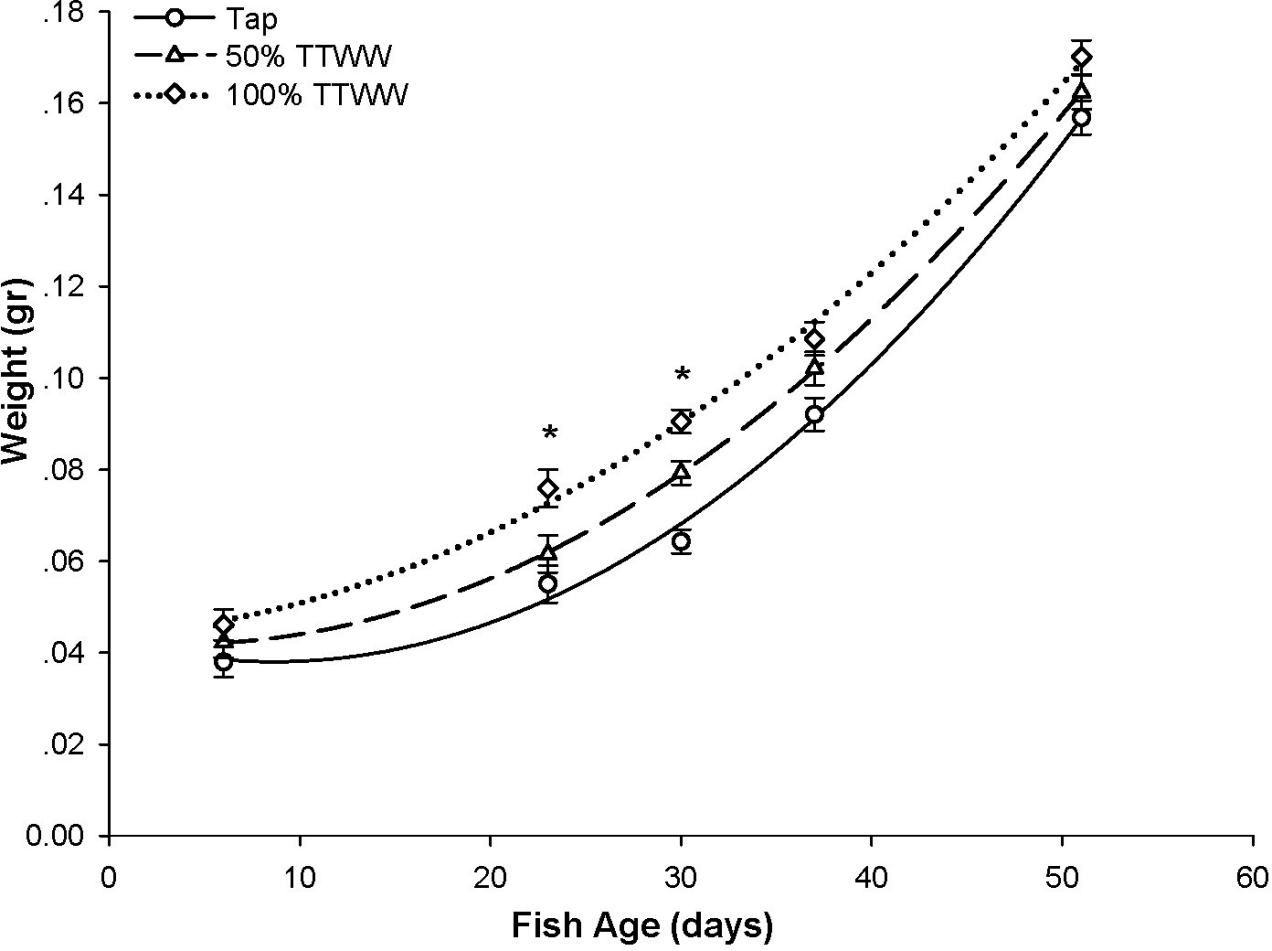
Somatic growth curves of fish in the different treatment groups before sexual maturity. Symbols represent mean body weight ± SE and lines represent a polynomial quadric (second order) regression curve fit. Asterisk indicates significant difference between 100% TTWW to tap, at the specific time point.

**Fig 3:**
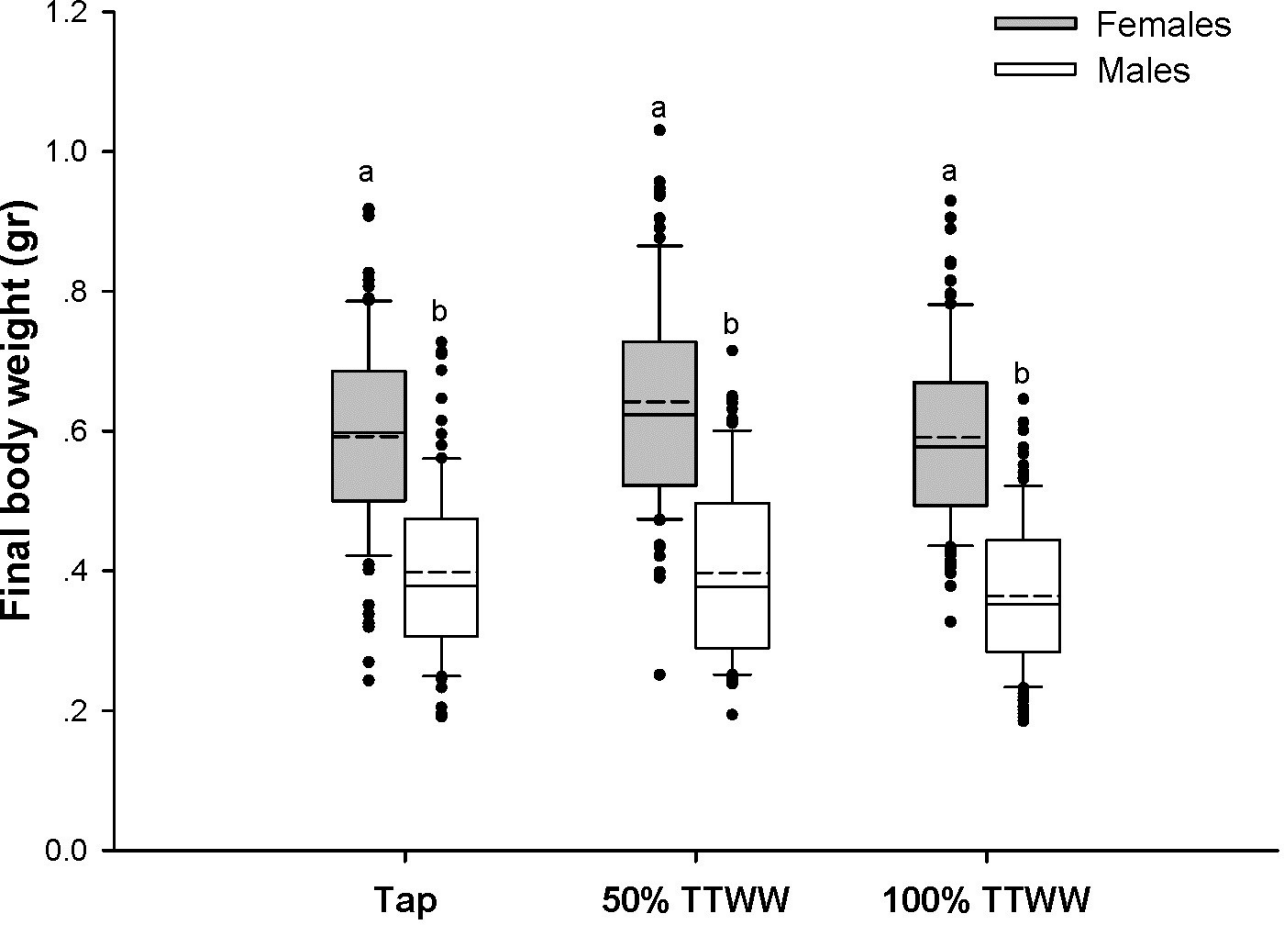
Final body weight (grams) of male and female guppies raised in different water treatments. Results presented as mean (dashed line), median (solid line), 25^th^ and 75^th^ percentiles (edges of boxes), 10^th^ and 90^th^ percentiles (whiskers), and outliers (dots). The a, b letters denote significant differences between males and females within each treatment groups (p < 0.05).

The fish somatic growth until day 51 was similar between treatment groups (Fig 2). Analyzing growth over time revealed no statistical differences between treatments. However, differences were found at specific time points, such as days 23 and 30, when fish grown in the 100% TTWW were significantly larger than those grown in tap water. Growth was individually measured on day 123 when the experiment was terminated. The final female body weights were slightly higher in the 50% TTWW (0.636 ± 0.015 gr) than in the tap water and 100% TTWW groups (0.583 ± 0.014 gr and 0.585 ± 0.014 gr, respectively). However, this difference was not statistically significant. The final male body weights averaged 0.397 ± 0.012, 0.399 ± 0.012 and 0.371 ± 0.012 gr in the tap, 50% TTWW and 100% TTWW treatment groups, respectively, with no significant differences between treatments (Fig 3).

Higher somatic growth of juvenile fish that were reared in treated wastewater was reported in several studies in different fish species. For example, *Rutilus rutilus* reared in ~80% secondary TWW for 300 days post-hatch were significantly larger than fish from the tap water control and the ~40% TWW-reared fish [60]. Similarly, growth of Nile tilapia fingerlings (*Oreochromis niloticus*) was elevated in 100% secondary TWW as compared to the control as determined over a nine-month culture period [14]. Feldlite et al. reported significantly higher somatic growth in common carp, hybrid Chinese carp, and tilapia reared in 100% TTWW for five months than in fish from the same species cultured in commercial fish ponds as a control [16]. African catfish (*Clarias gariepinus*) fingerlings cultured in 100% TWW from a stabilization pond exhibited elevated growth compared to the control fingerlings, which were cultured in a commercial pond over a 29-week period [61]. Other studies did not find differences between fish growth in wastewater and in a freshwater control. For example, juvenile high-black crucian carp (*Carassius auratus*) reared in 100% TTWW for 141 days [62] and *Rutilus rutilus* fry (30 days post-hatch) exposed for six months to 100% secondary or tertiary TWW [63] did not grow differently from freshwater-reared controls. Interestingly, to the best of our knowledge, there are no publications that suggest reduced growth due to culture in TWW.

The elevated growth reported in TWW by some of the studies may raise the assumption that residual steroid hormones in the water may have induced this effect. It should be noted that fish growth was previously reported to be promoted by diverse reproductive steroids at low concentrations [64,65], and the illegal use of steroids in an attempt to promote growth was reported in aquaculture farms [66]. In guppies, somatic growth in males, unlike females, typically ceases after two or three months, and only exposure to estrogenic compounds enables further growth [67]. Steroid hormones were measured in the monthly collected TTWW samples (Table 1), yet there was no measurable difference in the sizes of male guppies between the control and treatment groups.

### Hepato-somatic index

No significant differences in HSI were found between treatment groups or genders. The mean HSI ratios (males and females together) were 2.12 ± 0.07, 2.11 ± 0.07 and 2.16 ± 0.07 in tap water, 50% TTWW and 100% TTWW, respectively. Similarly, no differences in the HSI were found in males or females of crucian carp exposed to different TTWW dilutions, ranging between 25 and 100% for 28 days [68]. Differently from our results, significantly elevated HSI values were reported in adult roach of both genders, after 28 days of exposure to 100% secondary TWW [69], and in adult fathead minnow males, after 28 days exposure to 100% TTWW as compared to the control and 50% TTWW [70]. According to Tyler et al. (2005) exposure to high concentrations of estrogens increases HSI, by enhanced vitellogenin synthesis in the liver of exposed fish in both genders. Nevertheless, they reported similar HSI values and similar vitellogenin induction in immature rainbow trout and mature roach exposed to 100% TTWW for 8 and 10 days, although the measured estrogenic activity of the TWW was 2–3 order of magnitude higher than the control water [71]. Diniz et al. demonstrated elevated vitellogenin synthesis in TWW-exposed fish, yet argued that the HSI may be affected by other compounds in the TWW rather than by estrogenic substances alone [72]. Study done in natural water bodies receiving treated wastewater and containing estrogens, revealed seasonal changes in the HSI, but these did not correlate to plasma vitellogenin concentrations [73], supporting the assumption that elevated vitellogenesis in the liver caused by exposure to estrogenic factors is not the only cause for changes in HSI. Elevated HSI in fish may indicate stress response to TWW exposure and the different contaminants it contains (estrogenic and non-estrogenic), resulting in elevated hepatic lipid content and altered activity, such as gluconeogenesis and glycolysis [74,75].

### Immunology

**Fig 4:**
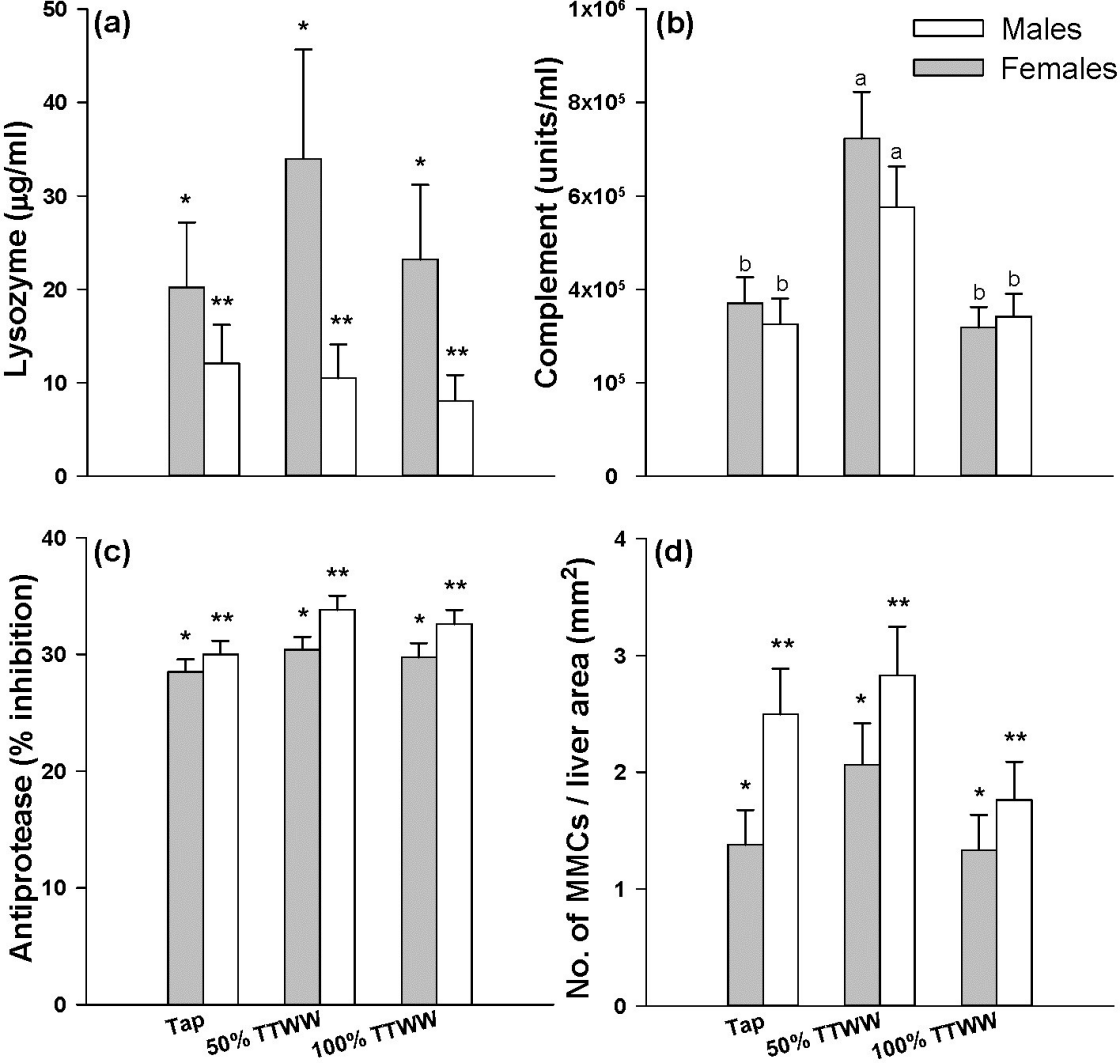
**Immunological parameters measured in analyses of whole body homogenates (a-c) or in histopathology examination (d).** (A) Lysozyme concentration (μg/mL), (B) Complement concentration (units/mL), (C) Anti-protease (% inhibition) and (D) ratio of the number of MMCs per liver area in mm^2^ in males and females from different treatment groups. a, b, different letters denote significant differences between treatment groups (p < 0.05); *, ** different signs denote significant differences between males and females (ANOVA, p < 0.05).

The innate immune system constitutes an essential part of the defense system in fish; therefore, humoral innate parameters, such as lysozyme and complement levels and antiprotease activity, can serve as indicators for overall immune function [47]. The potential effect of OMPs and heavy metals on fish’s immune function has scarcely been investigated, and the reported effects caused by exposure of fish to one or several heavy metals or OMPs were inconclusive, with reports ranging from immune suppression to immune activation [76,77]. In the present study, complement activity by the alternative pathway was significantly higher in fish cultured in 50% TTWW than in fish cultured in tap water and 100% TTWW (averages of 161,309 vs. 86,808 and 82,431 units/mL of body homogenate, respectively; Fig 4B). This was the only immunological parameter affected by the treatment. Interestingly, gender affected lysozyme and antiprotease activity, with lysozyme being significantly higher in females and antiprotease activity significantly higher in males (Fig 4A and 4C).

It is interesting to note that the TTWW had a pronounced brownish coloration, which may be attributed to the accumulation of humic substances that are the degradation products of organic compounds, typically of this brownish color [78]. These compounds are known to be present in recirculating aquaculture systems, originating from organic material that is present in these systems in a separate compartment from the fish culture tank. Recently, these substances were measured and characterized in a zero discharge recirculating aquaculture system and were shown to be present both in the water and in the fish’s blood [79]. A range of positive properties were attributed to humic substances in the water of cultured fish, including a protective effect from contaminants such as heavy metals, ammonia and nitrite [80–82], as well as a negative effect on fish pathogens, including species of fungi, bacteria and parasites [83–85]. Moreover, an immunostimulatory effect of humic substances was recently identified in common carp (*Cyprinus carpio*) and in guppies [85,86]. It is possible that the presence of humic substances in the water affected the fish’s immune function and could explain the higher lysozyme and complement activity in the 50% TTWW group. Further investigation of the occurrence and concentration of these compounds in TWW can be applied to test this assumption.

### *Tetrahymena* mortality

**Fig 5:**
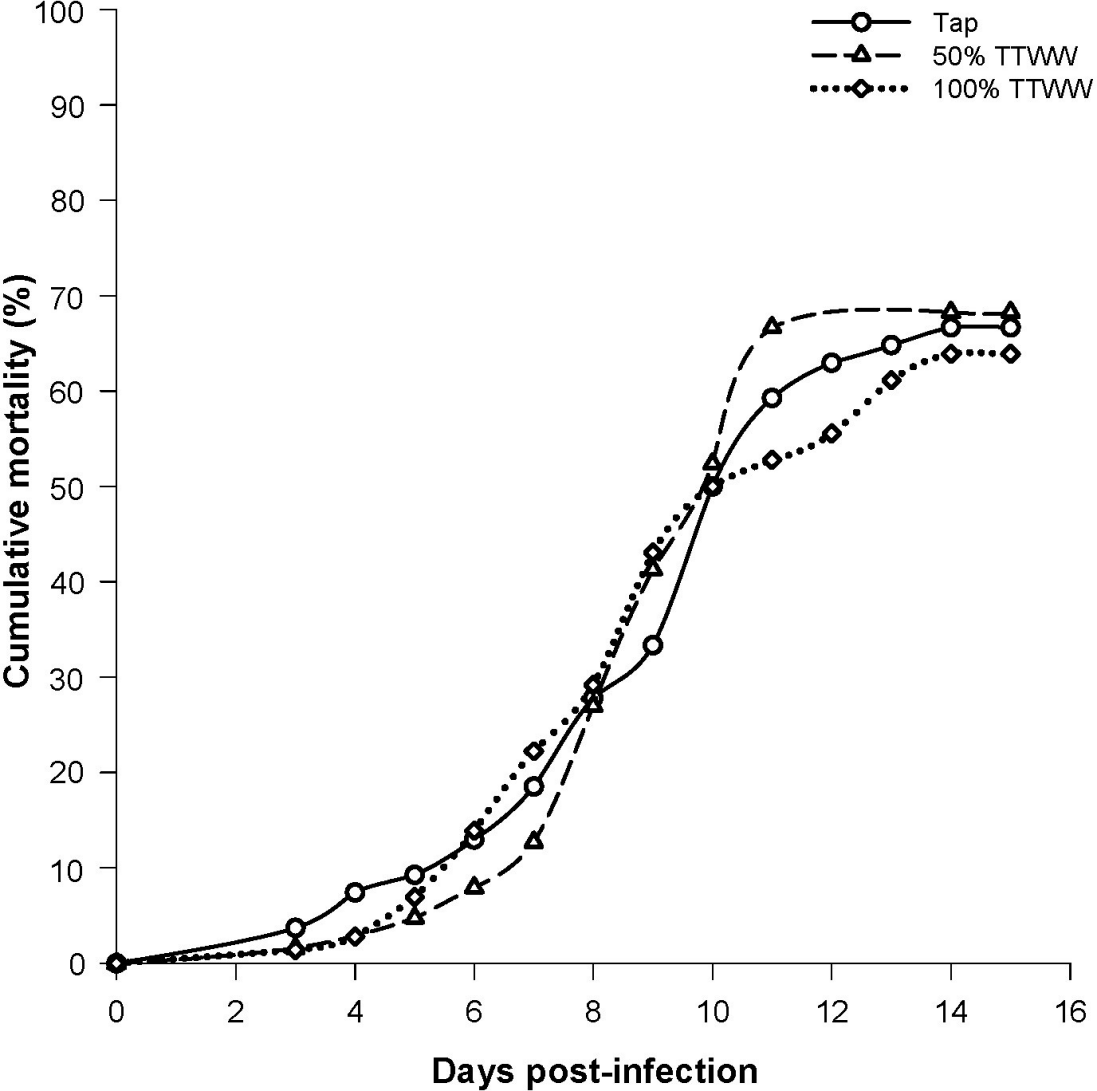
Cumulative mortality of guppies raised in different water groups, following infection with *Tetrahymena* sp.

Infection with *Tetrahymen*a resulted in mortality with a similar rate and total level in all treatment groups. At 15 days post-infection, mortality reached 67 ± 6% in tap water, 68 ± 9% in 50% TTWW and 64 ± 9% in TTWW, with no significant differences between treatment groups (Fig 5). *Tetrahymena* sp. is a ciliated protozoan and a highly pathogenic fish parasite, with a particular predisposition in guppies[87]. Susceptibility to infection increases under poor water quality, since *Tetrahymena* sp. is a saprophyte organism, and thus high loads of organic matter, ammonia and other nutrients in the water enable it to thrive, while the same conditions induce stress in fish[88]. Although TWW contains higher concentrations of organic matter and other nutrients than freshwater, it appears that the differences did not affect the parasite or the outcome of the challenge infection. As is evident from analysis of immune function and growth, the fish were not weakened by the water conditions, and evidently, their susceptibility to pathogenic infection did not increase. *Tetrahymena* was found to be sensitive to different pharmaceutical such as β-blockers and NSAIDs, and is therefore considered a suitable bioindicator of environmental pollution[89]. Similar mortality rates express similar Tetrahymena activity and can suggest that OMPs from the TTWW did not affect its activity, possibly due to their low concentrations.

### Heavy metals and PCBs in fish tissues

The concentrations of measured heavy metals in all samples were lower than the limit of detection (LOD) or the limit of quantification (LOQ) of the method, and much lower than the EU maximal permitted levels (Table 2). Similarly, PCBs and OCs in WBHs were below the detection limit (5 μg/Kg for PCBs and 1.5 μg/Kg for OCs), and much lower than the EU maximal permitted level of PCBs in fresh water fish muscle of 125 μg/Kg wet weight [90].

**Table 2:** Heavy metals measurements in fish pools from each aquarium. Each pool consists of 18–41 fish (whole body excluding the liver), total of four aquaria per treatment group. Concentrations are presented as ppb.

Heavy Metal	LOD	LOQ	Maximal permitted level*	Measured level											
				Tap				50% TTWW				100% TTWW			
				1	2	3	4	1	2	3	4	1	2	3	4
Cd	5	25	50^a^^,^^b^	N.D.	N.D.	N.D.	N.D.	N.D.	<LOQ	N.A.	N.D.	<LOQ	N.D.	N.D.	N.D.
As	100	500	1000^a^	<LOQ	<LOQ	<LOQ	<LOQ	<LOQ	<LOQ	N.D.	<LOQ	<LOQ	<LOQ	<LOQ	<LOQ
Pb	50	150	300^a^; 200^b^	N.D.	N.D.	N.D.	N.D.	<LOQ	N.D.	N.D.	N.D.	N.D.	<LOQ	N.D.	<LOQ
Hg	25	100	500^a^^,^ ^b^	<LOQ	<LOQ	<LOQ	<LOQ	<LOQ	<LOQ	<LOQ	<LOQ	<LOQ	<LOQ	<LOQ	<LOQ

N.D.–Not detected, values lower than limit of detection (LOD).

<LOQ: values between the limit of detection and the limit of quantification (LOQ).

*maximal permitted levels in food fish muscle

^a^European Commission (2006)

^b^FAO (2003)

Low values of heavy metals were also reported previously by Feldlite et al. who tested common carp (*Cyprinus carpio*), tilapia hybrid (*Oreochromis niloticus×O*. *aureus*), and hybrid Chinese carp (a hybrid of silver carp *Hypophthalmichthys molitrix* and bighead carp *Aristichthys nobilis*), which were reared in TTWW for five months and in secondary TWW for two years. Results revealed no detectable levels of Cd, As, Pb or Hg in fish flesh (excluding the skin). Some of these heavy metals were found only in the liver and bone (Cd in carp and tilapia) or only in the bone (Pb in carp) of fish grown in secondary TWW[16]. Cuevas-Uribe and Mims reported no detectable cadmium and lead in the muscle of paddlefish (*Polyodon spathula*) and hybrid striped bass (*Morone chrysops x M*. *saxatilis*) cultured in TTWW for 90 days. Mercury and selenium were detected in these fish in the same study, but at substantially lower levels than those permitted by the Food and Drug Administration (FDA) action limits[21]. Similarly, Adhikari et al. reported that the concentrations of lead, cadmium, chromium, copper and zinc in the flesh of one-year-old rohu (*Labeo rohita*), catla (*Catla catla*), mrigal (*Cirrhinus mrigala*), tilapia (*Oreochromis mossambicus*) and common carp (*Cyprinus carpio*), raised in the world's largest wastewater-fed aquaculture operation in Kolkata (India)[91], were far below the maximal permitted level set by the WHO/FAO. Interestingly, Aich et al. reported that metals accumulation in guppy whole body samples was lower in prolonged exposure (4 *vs*. 15 days) to tannery effluent, collected from East Calcutta Wetland Ecosystems. In addition, out of 6 measured metals (Pb, Cr, Mn, Fe, Cu and Zn) only Cr was significantly higher in the exposed fish samples in comparison to the control; Pb was lower than the detection limit in all treatment groups, and the rest of the measured metal’s concentrations were not significantly higher than the control [92].

The risk of heavy metal bioaccumulation in fish raised in wastewater-fed aquaculture is therefore considered to be low, as evidently heavy metal concentrations in fish flesh rarely exceed the maximal permitted levels by the FAO/WHO Codex Alimentarius Commission [93,94]. It should be noted that the heavy metal concentration in TWW is regularly monitored and highly regulated, and the maximal permitted levels of heavy metals in TWW for agricultural irrigation are low [95–97]. Thus, the chances of elevated levels of these compounds in municipal WWTPs are slim. In addition, heavy metal removal is highly correlated with total suspended solids (TSS) removal; therefore, a high removal efficiency (> 75%) of heavy metals can be achieved in most of the WWTPs by a TSS removal efficiency of > 90% [98]. These factors can help in keeping the risks from heavy metals in TTWW used for aquacultural purposes to a minimum.

### Histopathology

Examination of fish livers revealed no significant differences in the occurrence of MMCs/liver area between tap water (1.90 ± 0.24), 50% TTWW (2.43 ± 0.27) and 100% TTWW (1.54 ± 0.22). It was found that the ratio of MMCs:liver area in males was marginally significant higher than in females (Fig 4D). However, Paula et al. reported that after exposure of female guppies to 1.8 mg/L glyphosate-based herbicide (50% of the 96 h LC_50,_) for 4, 8, 12 and 24 hours, there was a significant increase in the occurrence of MMCs in the 24 hours group. They suggested that the activation of the immune system is the cause for this time-dependent response[50]. Elevated appearance of MMCs in the liver following exposure to TWW containing xenobiotics was reported in several other studies [48,49].

**Fig 6:**
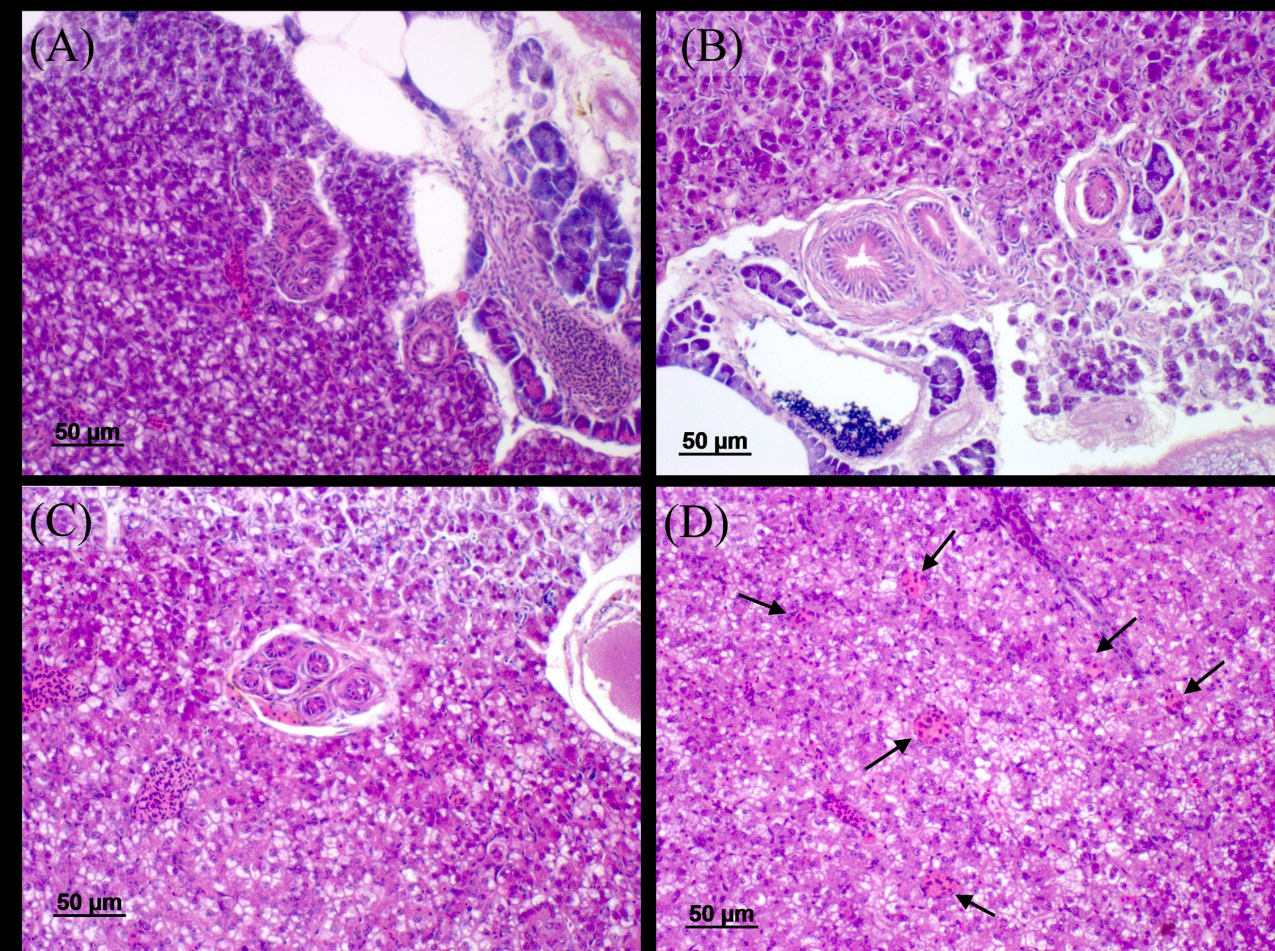
Histological sections of liver tissue. Bile duct hyperplasia in guppies reared in (A) tap water, (B) 50% TTWW and (C) 100% TTWW; (D) enlarged melanomacrophage centers (MMCs, arrows) in the liver of a fish grown in the tap treatment. MMCs were seen in the livers of fish from all treatment groups.

Although not affected by treatment, bile duct hyperplasia was observed in fish from the current study, at rates of 20.5 ± 6.5, 15 ± 5.7 and 18.4 ± 6.4% in tap water, 50% TTWW and 100% TTWW, respectively (Fig 6A–6C), but with no significant differences between treatment groups. Escher et al. described bile duct hyperplasia in the livers of brown trout (*Salmo trutta*) kept in moderately polluted river water and in water containing 5% of TWW, but not in fish raised in tap water[99]. According to Wester and Canton, in the exposure of guppies to MeHg at concentrations ranging from 1–10 μg/L for one month, bile duct hyperplasia was observed with increased frequency, starting from a concentration of 3.2 μg/L MeHg. However, exposure to concentrations of 1–5.6 μg/L for three months resulted in bile duct hyperplasia only in fish exposed to 5.6 μg/L of MeHg [100]. Although bile duct hyperplasia may be associated with exposure to contaminants, it can also emerge due to a parasitic infection [101]. In the present study, bile duct hyperplasia was found in fish from all treatment groups at similar rates, and therefore, this observed condition may be more related to parasitic infections (possibly *Gyroductilus turnbulii*, which was diagnosed during the trial) rather than to contaminant exposure.

**Fig 7:**
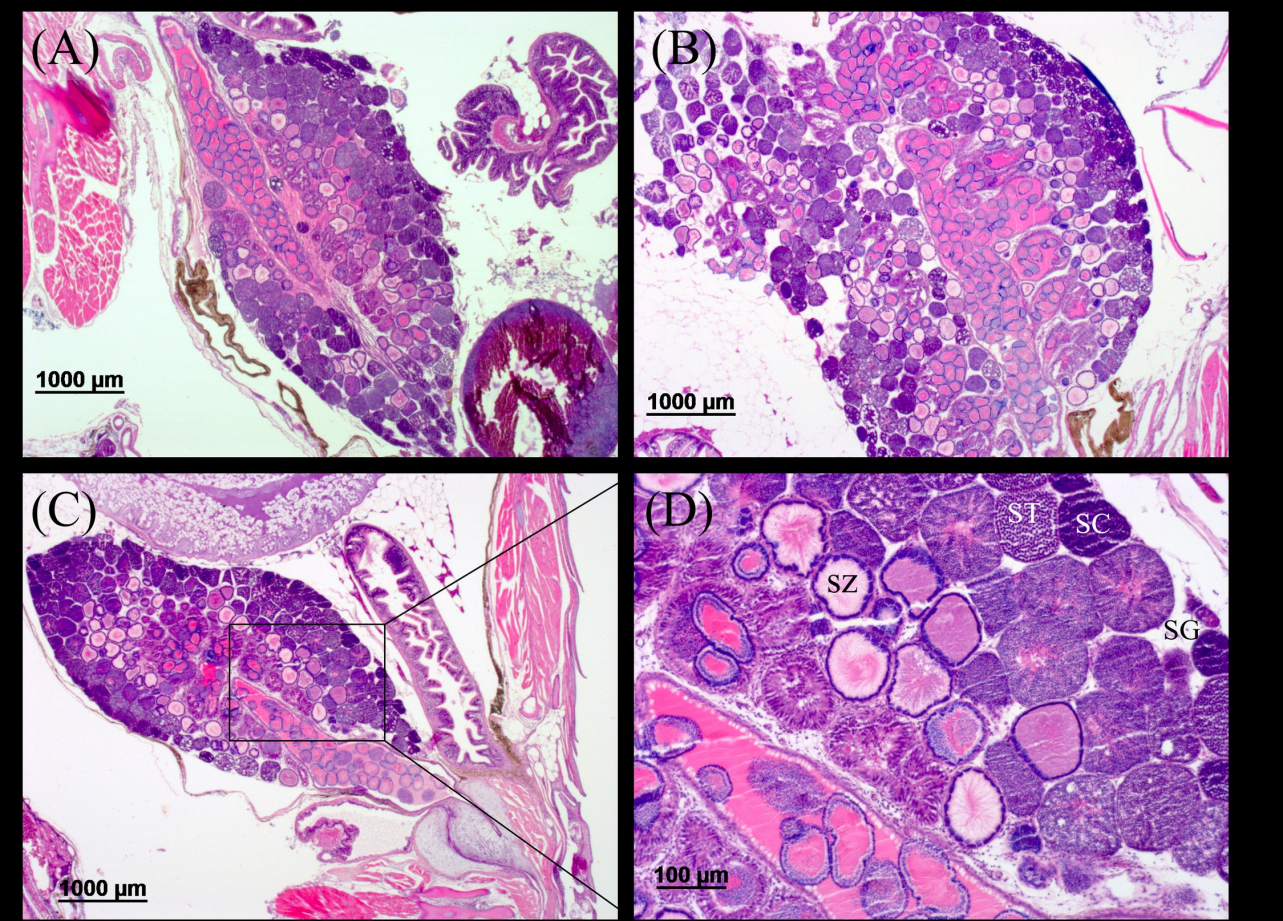
Longitudinal section of male gonads from different treatment groups. (A) tap, (B) 50% TTWW and (C) 100% TTWW; (D) testis demonstrating sperm duct (arrow) and all developmental stages of sperm cells: spermatogonia (SG); spermatocytes (SC); spermatids (ST); and spermatozoa (SZ).

**Fig 8:**
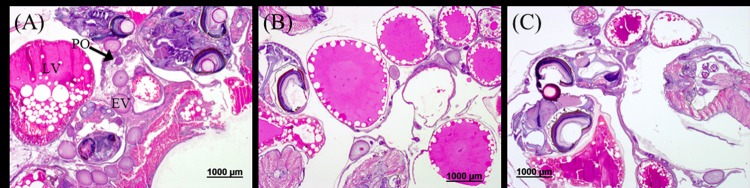
Sections of female ovaries from different treatment groups. (A) tap, (B) 50% TTWW and (C) 100% TTWW, showing embryos and oocytes at different stages of maturity: perinucleolar oocyte (PO), early vitellogenic oocyte (EV), and late vitellogenic oocyte (LV).

According to a histological analysis of male and female gonads, no differences were observed between the treatment groups. Exposure to TTWW at 50% and 100% did not seem to affect oocytes or testis maturation. Sperm cells at all developmental stages (spermatogonia, spermatocytes, spermatids and spermatozoa) were present in males raised in the different water treatments at similar levels (Fig 7A–7D), and oocytes and developing embryos were present in females from all treatment groups (Fig 8A–8C). Surprisingly, the single male with gonads containing both testis and oocytes was observed in the control (tap) group (S1 Fig). It is important to note that at the time of analysis, age of about 4 months, fish were sexually mature and at the stage that reproduction is known to occur in this species, which is 2–4 months of age. A similar observation was reported in African catfish reared in TWW for six months, in which mature gonads were detected in males and females from all ponds, and similar histological scores were found in male and female gonads from a WWTP stabilization pond in comparison to the control pond [102]. However, quite a large number of studies have reported on substantial alterations in male and female gonad development that were induced by exposure to TWW. Nielsen and Baatrup reported a significant reduction in the volume of early stage sperm cells (spermatocytes) in male guppies exposed to 17β-estradiol (10 and 50 ng L^-1^) for 3.5 months from birth. In the same study, exposure to 17α-ethinylestradiol (10, 50 and 200 ng L^-1^) revealed a dose-related decrease in sperm cell maturation [103]. Other studies have reported that exposure to estrogens led to the feminization of males. Roach exposed to 0, 15.2, 34.8 and 78.7% secondary- TWW (diluted with tap water) for 300 days exhibited a dose-related effect of male feminization [60]. The risk of the effect on sexual differentiation due to culture in TWW appears to vary between studies, but it is evident that the major concern is with respect to the effect on natural fish populations and the implication on ecological balances and species prosperity. In aquaculture, where fish are intended for harvesting, such physiological alterations are not a concern. Still, the presence of micropollutants originating from the TWW in the fish’s flesh should be further investigated and thoroughly analyzed. Although not regulated in food fish at present, they could pose a considerable health risk and must be examined before the culture of fish in TWW can be permitted as a common practice.

### Conclusions

In the current study, we examined the effect of tertiary treated wastewater on *Poecilia reticulata* (guppy) growth and health. The results revealed that TTWW did not affect the fish’s growth, immune function or disease resistance. Also, there was no evidence for associated histopathological changes. Heavy metal accumulations in the fish were below the LOQ, and far below the common maximal permitted levels in fish muscle according to the EU, FAO, and WHO standards. In addition, PCBs and OCs were not detected in fish tissues. The lack of detectable heavy metals, PCBs and OCs, which are the regulated parameters in fish for human consumption, suggests that these fish meet the current standards of consumer safety. The results of this study are in line with previous studies that examined the feasibility of TWW-fed aquaculture and indicate that fish culture in TTWW may be an applicable practice. While our measurements covered the regulated parameters for edible fish, further study is needed, particularly in the direct measurements of OMP bioaccumulation in fish flesh. In addition, the characteristics, standards and quality of treated wastewater are not universal; instead, they are dependent on different variables and should be taken into account on a national level and even a site level before permitting a certain water source to be used for fish production.

## Supporting information

S1 Table Concentrations (ng/L) of all the tested OMPs in tap water and TTWW in four separate samples that were collected during the trial (sample collection dates are indicated). (DOCX)

S1 File Statistical analyses information. Test types, results and statistical significance.
(DOCX)

S2 File Full data of all measured parameters and analyses performed in this study. (XLSX)

S1 Fig Histological section of a male guppy from the tap water treatment group that had both male and female gonads in a single individual. (A); showing testis with sperm cells at all developmental stages (B), and oocytes at different stages of maturation and spermatocytes (arrow, C).
(TIF)
