# Supplementary material for: The effect of tertiary treated wastewater on fish growth and health: Laboratory-scale experiment with Poecilia reticulata (guppy)
Source: PLoS One. 2019 Jun 11;14(6):e0217927. doi: 10.1371/journal.pone.0217927 (PMC6559704; doi:10.1371/journal.pone.0217927)
Supplement: S1 Table — (DOCX) [file pone.0217927.s001.docx]

| **Analytes** | **Method** | **Blank** | **Tap** | | | |  | **TTWW** | | | |  | |  |
| --- | --- | --- | --- | --- | --- | --- | --- | --- | --- | --- | --- | --- | --- | --- |
|  |  |  | **9-Dec-2015** | **6-Jan-2016** | **20-Jan-2016** | **2-Feb-2016** |  | **9-Dec-2015** | **6-Jan-2016** | **20-Jan-2016** | **2-Feb-2016** | |  | |
| Acesulfame | Direct Injection | < 15 | < 14 | < 11 | < 10 | < 12 |  | 261 | 2870 | 8860 | 7270 | |  | |
| Atenolol | Direct Injection | < 8.4 | < 5.7 | < 5.6 | < 5.4 | < 5.3 |  | 146 | 289 | 214 | 173 | |  | |
| Atrazine | OSPE | < 20 | < 28 | < 43 | < 31 | < 34 |  | < 37 | < 65 | < 42 | < 57 | |  | |
| Benzophenone | Direct Injection | < 100 | < 75 | < 80 | < 77 | < 77 |  | 196 | < 82 | 93 | 185 | |  | |
| Benzotriazole | OSPE | < 140 | < 240 | < 190 | < 160 | < 150 |  | 238 | 352 | 442 | 448 | |  | |
| Caffeine | Direct Injection | < 9.8 | < 9.4 | 15 | < 9.7 | < 9.9 |  | 104 | 730 | 640 | 199 | |  | |
| Carbamezapine | Direct Injection | < 13 | < 9.8 | < 12 | < 11 | < 11 |  | 1050 | 1810 | 1560 | 1350 | |  | |
| Clofibric Acid | OSPE | < 9.5 | < 7.9 | < 5.4 | < 5.3 | < 5.4 |  | < 8.7 | < 12 | < 10 | < 13 | |  | |
| DEET | Direct Injection | < 12 | < 10 | < 11 | < 11 | < 11 |  | < 11 | < 13 | < 14 | < 13 | |  | |
| Diclofenac | OSPE | < 7.5 | < 3.8 | < 5.0 | < 3.5 | < 4.4 |  | 82 | 654 | 729 | 628 | |  | |
| Diphenhydramine | Direct Injection | < 11 | < 8.2 | < 8.5 | < 8.7 | < 8.3 |  | 116 | 598 | 609 | 658 | |  | |
| Diltiazem | OSPE | < 3.7 | < 11 | < 9.8 | < 9.5 | < 9.7 |  | 43 | 19 | < 8.1 | < 8.3 | |  | |
| Fluoxetine | OSPE | < 14 | < 14 | < 28 | < 21 | < 18 |  | < 32 | < 49 | < 30 | < 35 | |  | |
| Gemfibrozil | OSPE | < 3.0 | < 1.5 | < 2.0 | < 1.4 | < 1.8 |  | < 1.8 | < 2.4 | < 1.7 | < 1.9 | |  | |
| Hydrochlorothiazide | OSPE | < 55 | < 48 | < 37 | < 31 | < 31 |  | 219 | 778 | 788 | 748 | |  | |
| Ibuprofen | OSPE | < 19 | < 21 | < 26 | < 20 | < 22 |  | < 29 | < 33 | < 29 | < 33 | |  | |
| Iohexol | Direct Injection | < 26 | < 57 | < 130 | < 97 | < 95 |  | 471 | 1110 | 1300 | 450 | |  | |
| Iopamidol | Direct Injection | < 13 | < 28 | < 63 | < 48 | < 47 |  | < 50 | < 78 | < 80 | < 67 | |  | |
| Iopromide | Direct Injection | < 26 | < 57 | < 130 | < 97 | < 95 |  | 175 | 208 | 3750 | 1060 | |  | |
| Meprobamate | Direct Injection | < 9.9 | < 9.5 | < 11 | < 9.6 | < 9.9 |  | 24 | 73 | 76 | 58 | |  | |
| Naproxen | OSPE | < 24 | < 20 | < 13 | < 13 | < 13 |  | < 22 | < 30 | < 26 | < 33 | |  | |
| PFBS | OSPE | < 8.2 | < 6.5 | < 6.9 | < 5.9 | < 7.0 |  | < 7.1 | < 8.0 | < 7.0 | < 7.0 | |  | |
| PFHpA | OSPE | < 4.1 | < 3.2 | < 3.5 | < 3 | < 3.5 |  | < 3.5 | < 4.0 | < 3.5 | < 3.5 | |  | |
| PFHxA | OSPE | < 8.2 | < 6.5 | < 6.9 | < 5.9 | < 7.0 |  | < 7.1 | < 8.0 | < 7.0 | < 7.0 | |  | |
| PFOA | OSPE | < 8.2 | < 6.5 | < 6.9 | < 5.9 | < 7.0 |  | < 7.1 | < 8.0 | < 7.0 | < 7.0 | |  | |
| PFOS | OSPE | < 51 | < 34 | < 41 | < 34 | < 29 |  | < 39 | < 41 | < 37 | < 54 | |  | |
| Primidone | OSPE | < 30 | < 35 | < 42 | < 34 | < 36 |  | < 65 | < 81 | < 75 | < 76 | |  | |
| Propranolol | OSPE | < 10 | < 8.0 | < 7.0 | < 6.8 | < 6.5 |  | 15 | 37 | 22 | 22 | |  | |
| Propylparaben | OSPE | < 10 | < 8.0 | < 7.0 | < 6.8 | < 6.5 |  | < 9 | <16 | < 13 | < 14 | |  | |
| Simazine | OSPE | < 8.2 | < 11 | < 17 | < 13 | < 14 |  | < 15 | < 26 | < 17 | < 23 | |  | |
| Sucralose | Direct Injection | < 44 | < 12 | < 7.2 | < 8.6 | < 9.2 |  | 9030 | 15900 | 16800 | 14100 | |  | |
| Sulfamethoxazole | Direct Injection | < 11 | < 8.5 | < 9.8 | < 9.6 | < 8.8 |  | 241 | 228 | 264 | 239 | |  | |
| TCEP | Direct Injection | < 11 | < 7.4 | < 8.3 | < 7.9 | < 8.2 |  | 153 | 189 | 166 | 186 | |  | |
| TCPP | Direct Injection | < 21 | < 15 | 61 | 140 | 65 |  | 1750 | 2830 | 4310 | 2050 | |  | |
| Triclocarban | Direct Injection | < 7.9 | < 6.4 | < 5.3 | < 5.3 | < 5.4 |  | < 5.3 | < 5.2 | < 5.2 | < 5.3 | |  | |
| Triclosan | Direct Injection | < 6.7 | < 7.9 | < 7.4 | < 7.4 | < 7.6 |  | < 7.7 | < 8.1 | < 8.0 | < 7.9 | |  | |
| Trimethoprim | OSPE | < 12 | < 15 | < 29 | < 21 | < 22 |  | 105 | 70 | 59 | 49 | |  | |
| **Estrogens** |  |  |  |  |  |  |  |  |  |  |  | |  | |
| Estrone |  | < 0.3 | < 0.3 | < 0.3 | < 0.3 | < 0.3 |  | 0.6 | 2.2 | 2.6 | 1.8 | |  | |
| 17b-Estradiol |  | < 0.3 | < 0.3 | < 0.3 | < 0.3 | < 0.3 |  | 1.1 | 1.6 | 1.0 | 0.9 | |  | |
| 17a-Estradiol |  | < 0.3 | < 0.3 | < 0.3 | < 0.3 | < 0.3 |  | < 0.3 | < 0.5 | < 0.4 | < 0.4 | |  | |
| Estriol |  | < 0.1 | < 0.2 | < 0.1 | < 0.1 | < 0.1 |  | < 0.3 | < 0.5 | < 0.5 | < 0.4 | |  | |
| 17a-Ethynylestradiol |  | < 0.5 | < 0.5 | < 0.6 | < 0.5 | < 0.5 |  | < 0.6 | < 0.9 | < 0.8 | < 0.7 | |  | |
| Bisphenol A |  | < 1.1 | 4.6 | 9.5 | 4.1 | 4.2 |  | 740 | 39.8 | 15.6 | 8.5 | |  | |
| **Progestogens and Androgens** |  |  |  |  |  |  |  |  |  |  |  | |  | |
| Progesterone |  | < 0.1 | < 0.2 | 0.47 | 1.1 | 0.8 |  | 2.2 | 2.2 | 1.7 | 1.2 | |  | |
| 17-Hydroxyprogesterone |  | < 0.5 | < 1.0 | < 0.9 | < 0.8 | < 0.8 |  | 3.0 | < 1.8 | < 1.7 | < 1.7 | |  | |
| Norethindrone |  | < 0.1 | < 0.2 | < 0.2 | < 0.2 | < 0.2 |  | < 0.3 | < 0.4 | < 0.4 | < 0.4 | |  | |
| Norgestrel |  | < 1.1 | < 2.1 | < 1.9 | < 1.5 | < 1.7 |  | < 3.2 | < 3.7 | < 3.5 | < 3.4 | |  | |
| Testosterone |  | < 0.3 | < 0.5 | < 0.5 | 0.4 | 0.7 |  | 2.6 | 3.8 | 3.5 | 2.2 | |  | |
| **Corticosteroids** |  |  |  |  |  |  |  |  |  |  |  | |  | |
| Aldosterone |  | < 0.3 | < 0.5 | < 0.4 | < 0.3 | < 0.4 |  | < 0.4 | < 0.5 | < 0.7 | < 0.7 | |  | |
| Betamethasone |  | < 0.1 | < 0.1 | < 0.1 | < 0.1 | 0.1 |  | 1.4 | 2.3 | 1.7 | 1.4 | |  | |
| Budesonide |  | < 0.3 | < 1.0 | < 0.6 | < 0.4 | < 0.4 |  | < 2.0 | < 2.3 | < 1.8 | < 2.0 | |  | |
| Clobetasol propionate |  | < 0.1 | < 0.1 | < 0.1 | < 0.1 | < 0.1 |  | < 0.5 | < 0.4 | < 0.4 | < 0.3 | |  | |
| Corticosterone |  | < 0.1 | < 0.1 | < 0.1 | < 0.1 | < 0.1 |  | < 0.1 | < 0.1 | < 0.1 | < 0.1 | |  | |
| Cortisone |  | < 0.3 | < 0.5 | < 0.4 | < 0.3 | < 0.3 |  | < 0.3 | < 0.5 | < 0.6 | < 0.5 | |  | |
| Deoxycorticosterone acetate |  | 0.5 | < 0.5 | < 0.5 | < 0.4 | 0.4 |  | 4.9 | 12.4 | 2.2 | < 1.0 | |  | |
| Fludrocortisone acetate |  | < 0.1 | < 0.4 | < 0.3 | < 0.2 | < 0.2 |  | < 1.0 | < 3.0 | < 3.0 | < 2.0 | |  | |
| Flumethasone |  | < 0.1 | < 0.1 | < 0.1 | < 0.1 | < 0.1 |  | < 0.1 | < 0.1 | < 0.1 | < 0.1 | |  | |
| Fluocinolone acetonide |  | < 0.3 | < 0.4 | < 0.3 | < 0.2 | < 0.3 |  | 5.0 | 1.5 | 64.4 | 33.1 | |  | |
| Fluocinonide |  | < 0.1 | < 0.2 | < 0.2 | < 0.2 | < 0.1 |  | < 1.0 | < 0.8 | < 0.8 | < 0.6 | |  | |
| Fluorometholone |  | < 0.3 | < 1.0 | < 0.6 | < 0.4 | < 0.4 |  | < 2.0 | < 2.3 | < 1.8 | < 2.0 | |  | |
| Fluticasone propionate |  | 0.2 | < 0.2 | < 0.2 | < 0.2 | < 0.1 |  | < 1.0 | 1.1 | 2.9 | 2.7 | |  | |
| Hydrocortisone |  | < 0.1 | 0.2 | < 0.1 | 0.1 | 0.1 |  | 0.4 | 0.5 | 0.5 | 0.3 | |  | |
| Methylprednisolone |  | < 0.3 | N.A | N.A | N.A | N.A |  | < 0.3 | < 0.4 | < 0.6 | < 0.6 | |  | |
| Prednisolone |  | < 0.3 | < 0.4 | < 0.3 | < 0.3 | < 0.3 |  | < 0.3 | < 0.5 | < 0.7 | < 0.6 | |  | |
| Prednisone |  | < 0.1 | < 0.2 | < 0.2 | 0.3 | 0.6 |  | 2.9 | 5.0 | 4.3 | 3.4 | |  | |
| Triamcinolone acetonide |  | < 0.3 | < 0.4 | < 0.3 | < 0.2 | < 0.3 |  | 1.8 | 4.0 | 87.5 | 22.9 | |  | |

N.A: not analyzed
