# Supplementary material for: The effect of tertiary treated wastewater on fish growth and health: Laboratory-scale experiment with Poecilia reticulata (guppy)
Source: PLoS One. 2019 Jun 11;14(6):e0217927. doi: 10.1371/journal.pone.0217927 (PMC6559704; doi:10.1371/journal.pone.0217927)
Supplement: S1 File — Test types, results and statistical significance. (DOCX) [file pone.0217927.s002.docx]

**Statistical analyses:**

The most appropriate statistical analysis was considered for each of the analyzed parameters, considering the experimental setup and the statistical requirements (validation of the assumptions etc.)

In our experimental setup the aquaria are the experimental units. Since the individual fish from the same aquarium are not independent, they were considered as sub-samples (pseudo-replication), increasing the precision of the experimental replicate (aquarium), thus increasing the power of the test. Therefore, when it was possible (according to the assumptions) - individuals' data were used in nested design ANOVA, and when normality failed – the means of the aquaria (plots) or other factor (gender) were used in a simpler model (e.g. 2-way ANOVA instead 3-way ANOVA).

**I. Parameter: Survival**

Test type: Gehan-Breslow (survival analysis)

Df = 2

P = 0.393

Statistics: 1.867

|  | n | events | censored | %censored |
| --- | --- | --- | --- | --- |
| Tap | 400 | 82 | 318 | 80 |
| 50%TTWW | 400 | 97 | 303 | 76 |
| 100%TTWW | 400 | 94 | 306 | 77 |

| Holm-Sidak (0.05) | statistics | p |
| --- | --- | --- |
| Tap vs. 50%TTWW | 1.575 | 0.0506 |
| Tap vs. 100%TTWW | 1.289 | 0.447 |
| 50% vs. 100% | 0.00615 | 0.937 |

**II. Parameter: Growth**

Growth was analyzed by two different tests, MANOVA and repeated measures ANOVA for split plot design

| Post-Hoc: Bonferroni |  |
| --- | --- |
| error between MSE | 0 |
| df | 9 |
| Tap vs. 50% | 1.000000 |
| Tap vs. 100% | 0.349471 |
| 50% vs. 100% | 1.000000 |

a. Test type: MANOVA

|  | Test | Value | F | effect df | error df | p |
| --- | --- | --- | --- | --- | --- | --- |
| Water treatment | Wilks | 0.098 | 2.191 | 10 | 10 | 0.116033 |
|  | Pillai's | 1.110 | 1.498 | 10 | 12 | 0.250665 |
|  | Hotellng | 7.059 | 2.824 | 10 | 8 | 0.077338 |
|  | Roy's | 6.744 | 8.093 | 5 | 6 | 0.012131 |

b. Test type: repeated measures ANOVA for split plot design

Mauchly's Sphericity Test: (Sphericity assumption)

| DF | Mauchly's Statistic | Chi-square | Pr > Chi² | Greenhouse-Geisser Epsilon | Huynt-Feldt Epsilon |
| --- | --- | --- | --- | --- | --- |
| 9 | 0.172732327 | 16.53578087 | 0.075 | 0.585 | 0.978 |

Result of the repeated measures ANOVA:

| Effect | df | SS | MS | F | p |
| --- | --- | --- | --- | --- | --- |
| Water treatment*fish age | 8 | 0.000448 | 0.000056 | 1.93 | 0.085598 |
| Error | 36 | 0.001044 | 0.000029 |  |  |
| Total |  | 0.108361 |  |  |  |

* Since 3 out of the 4 MANOVA tests and the repeated measures ANOVA for split plot design revealed no overall significant difference in the growth rate, we concluded that the differences between the curves were not significant (only in few time points).

**III. Parameter: Final body weight** (males and females' data were analyzed separately due to the known differences in body weight)

Test types:

Females – 2-way nested ANOVA (factor 1 – water treatment, factor 2 – aquarium no., data – individual weights nested in plot after data transformation square root).

|  | Effec (F/R)t | SS | df | MS | F | p |
| --- | --- | --- | --- | --- | --- | --- |
| Water treatment | Fixed | 0.1742 | 2 | 0.0871 | 0.850 | 0.458828 |
| Aquarium rep. (Water treatment) | Random | 0.9507 | 9 | 0.1056 | 5.592 | 0.000000 |
| Error |  | 5.3082 | 281 | 0.0189 |  |  |

|  | |
| --- | --- |
|  |  |
|  |  |
|  |  |
|  |  |
|  |  |

Data after back transformation (including tap 3)

|  | mean | SE | N |
| --- | --- | --- | --- |
| Tap | 0.610 | 0.016 | 102 |
| 50% TTWW | 0.633 | 0.016 | 89 |
| 100% TTWW | 0.584 | 0.012 | 102 |

Post-Hoc: Tukey HSD (within treatments)

|  | |  | Tap |  |  |  | 50%TTWW |  |  |  | | 100%TTWW | |  | |  |
| --- | --- | --- | --- | --- | --- | --- | --- | --- | --- | --- | --- | --- | --- | --- | --- | --- |
|  |  | 1 | 2 | 3 | 4 | 1 | 2 | 3 | 4 | 1 | 2 | | 3 | | 4 | |
| Tap | 1 |  | 1.000000 | 0.000984 | 1.000000 |  |  |  |  |  |  | |  | |  | |
|  | 2 | 1.000000 |  | 0.000276 | 0.999996 |  |  |  |  |  |  | |  | |  | |
|  | 3 | 0.000984 | 0.000276 |  | 0.000938 |  |  |  |  |  |  | |  | |  | |
|  | 4 | 1.000000 | 0.999996 | 0.000938 |  |  |  |  |  |  |  | |  | |  | |
| 50%TTWW | 1 |  |  |  |  |  | 0.462556 | 0.991234 | 0.065447 |  |  | |  | |  | |
|  | 2 |  |  |  |  | 0.462556 |  | 0.981769 | 0.999687 |  |  | |  | |  | |
|  | 3 |  |  |  |  | 0.991234 | 0.981769 |  | 0.570770 |  |  | |  | |  | |
|  | 4 |  |  |  |  | 0.065447 | 0.999687 | 0.570770 |  |  |  | |  | |  | |
| 100%TTWW | 1 |  |  |  |  |  |  |  |  |  | 1.000000 | | 0.993202 | | 0.901672 | |
|  | 2 |  |  |  |  |  |  |  |  | 1.000000 |  | | 0.999554 | | 0.625253 | |
|  | 3 |  |  |  |  |  |  |  |  | 0.993202 | 0.999554 | |  | | 0.099495 | |
|  | 4 |  |  |  |  |  |  |  |  | 0.901672 | 0.625253 | | 0.099495 | |  | |

According to a Post-Hoc test within each treatment, final body weight of females from Tap 3 was significantly larger than other Tap aquaria, and therefore we decided to remove this aquarium, and to perform the analysis without data of females from Tap3:

|  | Effec (F/R)t | SS | df | MS | F | p | Observed power (alpha=0.05) |
| --- | --- | --- | --- | --- | --- | --- | --- |
| Water treatment | Fixed | 0.0667 | 2 | 0.0334 | 1.563 | 0.267718 | 0.240468 |
| Aquarium rep. (Water treatment) | Random | 0.1697 | 8 | 0.0212 | 2.734 | 0.006500 | 0.933160 |
| Error |  | 2.0802 | 268 | 0.0078 |  |  |  |

|  | |
| --- | --- |
|  |  |
|  |  |
|  |  |
|  |  |
|  |  |

Data after back transformation (without tap 3)

|  | mean | SE | N |
| --- | --- | --- | --- |
| Tap | 0.583 | 0.014 | 88 |
| 50% TTWW | 0.636 | 0.015 | 89 |
| 100% TTWW | 0.585 | 0.013 | 102 |

Males – 1-way ANOVA (factor 1 – water treatment, data – aquaria averages)

F=1.718

P=0.233

N total = 12

**IV. Parameter: Mortality following infection with Tetrahymena**

Test type: Gehan-Breslow (survival analysis)

df = 2

p = 0.989

| Holm-Sidak (0.05) | statistics | p |
| --- | --- | --- |
| Tap vs. 50%TTWW | 0.036 | 0.997 |
| Tap vs. 100%TTWW | 0.0037 | 0.998 |
| 50% vs. 100% | 0.0000572 | 0.994 |

Statistics: 0.0211

|  | n | events | censored | %censored |
| --- | --- | --- | --- | --- |
| Tap | 54 | 36 | 18 | 33 |
| 50%TTWW | 63 | 43 | 20 | 32 |
| 100%TTWW | 72 | 46 | 26 | 36 |

**V. Parameter: Complement**

Test type: 3-way nested ANOVA [factor 1 – water treatment, factor 2 – aquarium no. (nested in water treatment), factor 3 – gender, data – individuals values after data transformation log10]

df = 2

F(2, 4.5703)=7.1727

p = 0.038934

N = 26 (Tap), 30 (50% TTWW), 31 (100% TTWW)

| Post-Hoc: Tukey HSD | |
| --- | --- |
| error between MSE | 0.058 |
| df | 72 |
| Tap vs. 50% | 0.000919 |
| Tap vs. 100% | 0.867382 |
| 50% vs. 100% | 0.000173 |

**VI. Parameter: MMCs in liver (histology)**

Test type: 3-way nested ANOVA [factor 1 – water treatment, factor 2 – aquarium no. (nested in water treatment), factor 3 – gender, data – individuals values after data transformation square root]. Post-Hoc: Tukey HSD.

|  | Effect | df | SS | MS | F | p |
| --- | --- | --- | --- | --- | --- | --- |
| Water treatment | Fixed | 2 | 1.9911 | 0.9956 | 1.47681 | 0.311785 |
| Aquarium no. (Water treatment) | Random | 7.5907 | 9 | 0.8434 | 2.76709 | 0.006137 |
| Gender | Random | 1 | 2.1957 | 2.1957 | 16.06550 | 0.056863 |
| Water treatment*Gender | Random | 2 | 0.2733 | 0.1366 | 0.4482 | 0.639997 |
| Error |  | 102 | 31.0894 | 0.3048 |  |  |

**VII. Parameter HSI, Lysozyme, Anti-protease:**

2-way ANOVA (factor 1 – water treatment, factor 2 – gender, data – averages of males and females separately from each aquaria). Post Hoc – Bonferroni.

df= 2 (Water treatment), 1 (Gender), 2 (Water treatment*gender)

|  | HSI | | Lysozyme(data transformation – log10) | | Anti-protease | |
| --- | --- | --- | --- | --- | --- | --- |
|  | F | p | F | p | F | p |
| Water treatment | 0.129 | 0.879397 | 0.85984 | 0.537681 | 1.868 | 0.183195 |
| Gender | 3.290 | 0.086428 | 20.49188 | 0.045495 | 6.944 | 0.016806 |
| Water treatment*Gender | 1.929 | 0.174187 | 0.53594 | 0.594172 | 1.266 | 0.305838 |
