# Supplementary figures and images for: The effect of tertiary treated wastewater on fish growth and health: Laboratory-scale experiment with Poecilia reticulata (guppy)
Source: PLoS One. 2019 Jun 11;14(6):e0217927. doi: 10.1371/journal.pone.0217927 (PMC6559704; doi:10.1371/journal.pone.0217927)

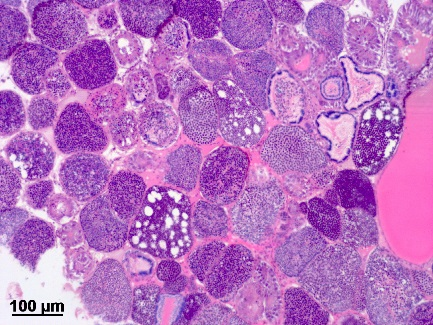

Supplement: S1 Fig — (A); showing testis with sperm cells at all developmental stages (B), and oocytes at different stages of maturation and spermatocytes (arrow, C). (TIF) [file pone.0217927.s004.tif]
